# Comparison of methods to detect differentially expressed genes between single-cell populations

**DOI:** 10.1093/bib/bbw057

**Published:** 2016-07-02

**Authors:** Maria K Jaakkola, Fatemeh Seyednasrollah, Arfa Mehmood, Laura L Elo

**Affiliations:** 1 Turku Centre of Biotechnology, University of Turku, Tykistökatu 6, Turku, Finland; 2 Department of Mathematics and Statistics, University of Turku, Turku, Finland

**Keywords:** single-cell, comparison, differential expression, RNA-seq, reproducibility

## Abstract

We compared five statistical methods to detect differentially expressed genes between two distinct single-cell populations. Currently, it remains unclear whether differential expression methods developed originally for conventional bulk RNA-seq data can also be applied to single-cell RNA-seq data analysis. Our results in three diverse comparison settings showed marked differences between the different methods in terms of the number of detections as well as their sensitivity and specificity. They, however, did not reveal systematic benefits of the currently available single-cell-specific methods. Instead, our previously introduced reproducibility-optimization method showed good performance in all comparison settings without any single-cell-specific modifications.

## Background

Gene expression profiling has traditionally focused on bulk populations of millions of cells, measured either using microarrays or next-generation sequencing technologies. Although an average expression level for each gene in the cell population can be sufficient in many applications, such as determining disease biomarkers, bulk RNA-seq analysis lacks the detail of cell-specific functionality. Consequently, with the rapid technological developments, single-cell RNA-seq (scRNA-seq) has quickly grown into a popular field of RNA-sequencing, enabling novel biological discoveries such as detecting novel cell types with distinct expression signatures, or understanding of the stochasticity of gene expression in a cell population [1–3].

To effectively use the scRNA-seq data, it is crucial to use appropriate tools for the data analysis. Although several sophisticated methods are already available for analyzing bulk RNA-seq data [4–6], their direct applicability to scRNA-seq data still remains largely unclear. In particular, scRNA-seq has its own specificities and challenges, a major challenge being how to distinguish technical and biological noise. This stems, on the one hand, from the low amount of starting material and, on the other hand, from inherent biological variability [7]. However, the number of measurements per group is typically considerably higher in scRNA-seq experiments than in bulk RNA-seq experiments, which can, at least to some extent, compensate the higher noise levels [3].

A common task in many single-cell studies is to detect differentially expressed (DE) genes between cell populations. There are already multiple methods that can be used for that purpose. Some of the methods are new and designed specifically for scRNA-seq data, some have been originally developed for bulk RNA-seq data, and some are more general. Recently, Kharchenko *et al*. [7] briefly compared some of the single-cell methods with their novel method presented in the article but there still remains a lack of a systematic comparison of the currently available tools for scRNA-seq data.

To address this need, we compared five representative statistical methods that detect DE genes between single-cell populations: (1) single-cell differential expression (SCDE) [7], (2) model-based analysis of single-cell transcriptomics (MAST) [8], (3) differential expression analysis for sequence count data (DESeq) [9], (4) Linear models for microarray and RNA-Seq data (Limma) [10] and (5) reproducibility-optimized test statistic (ROTS) [11–13]. SCDE and MAST are specifically designed for single-cell data, DESeq and Limma are methods developed originally for conventional bulk RNA-seq data and ROTS is a general statistical method, which learns the appropriate test statistic directly from the data. To systematically assess whether the methods designed specifically for scRNA-seq data perform better than the methods developed for bulk RNA-seq data or the more general methods, three diverse comparisons were considered. The first one was similar to the one in [7]: there are two independent data sets including measurements from the same two cell types [14, 15] and the methods are expected to find the same genes as significant in both data sets. In the other two comparisons, we investigated reproducibility of the detections, false positive findings and the effect of the number of measured cells on the results using two recently published large single-cell data sets by Kowalczyk *et al*. [16] and Björklund *et al*. [17].

## Methods to detect DE genes

In this section, we shortly introduce the five tested methods with both a general summary and by a brief description of the mathematical basis of the methods. Detailed explanations are available in the original publications [7–11]. The differential expression methods were applied following the instructions and recommendations of their respective software packages. Genes with false discovery rate (FDR) <0.05 were considered as DE. To make the comparisons fully reproducible, we provide the codes and parameters from our analyses in the Supplementary Code. The methods to be tested were first selected on the basis of the comparison by Kharchenko *et al*. [7]. MAST is essentially the same method as Single Cell Assay (SCA) tested in [7]; CuffDiff2 was excluded from the study based on poor performance in previous comparisons [6, 7]. Two additional methods, Limma and ROTS, were further added to this study to investigate whether scRNA-seq-specific methods perform significantly better than more general methods for detecting differentially behaving elements between two groups. The following R packages were used:

- SCDE (v. 1.99.0) was downloaded from

http://hms-dbmi.github.io/scde/package.html

- MAST (v. 0.931) was downloaded from

https://github.com/RGLab/MAST

- DESeq (v. 1.20.0) is available in

Bioconductor (http://www.bioconductor.org)

- Limma (v. 3.24.15) is available in

Bioconductor (http://www.bioconductor.org)

- ROTS (v. 0.99.9) is available in

Bioconductor (http://www.bioconductor.org)

### Single-cell differential expression (SCDE)

The SCDE method is a recently introduced novel method designed for single-cell data analysis [7]. SCDE analysis consists of three main steps: data filtering, fitting an error model and testing for differential expression. In the data filtering step, both genes that are not expressed in any of the tested cells and cells that show poor coverage over measured genes are excluded from the analysis.

When comparing two subgroups of cells *S* and *G* to each other, the probability of fold expression difference of *f* in gene *g* is determined by

(1) $$p_{g}(f)=\sum_{x\in X}p_{Sg}(x)\cdot p_{Gg}(fx),$$

where *X* is the range of valid expression levels and $p_{Sg}(x)$ is the posterior probability of gene *g* being expressed at an average level *x* in subpopulation *S* (*p_Gg_* is defined similarly). In Bayesian approach, the probability $p_{Sg}(x)$ is defined as an expected value (*E*):

(2) $$p_{Sg}(x)=E\left[\prod_{c\in B}p_{g}(x|r_{c},\Omega_{c})\right],$$

where *B* is a bootstrap sample of *S* and $p_{g}(x|r_{c},\Omega_{c})$ is the posterior probability of gene *g* being expressed at level *x* in cell *c* conditioned on observed expression level *r_c_* and fitted error model Ω_*c*_ for cell *c*. The posterior probability can be further written as

(3) $$p_{g}(x|r_{c},\Omega_{c})=p_{d}(x)p_{poisson}(x)+(1-p_{d}(x))p_{NB}(x|r_{c}),$$

where $p_{d}(x)$ is the probability of dropout in cell *c* when a gene is expressed at an average level of *x*, and *p_poisson_* and *p_NB_* are probabilities of observing expression magnitude of *x* in case of a dropout (Poisson) or successful amplification (negative binomial NB), with the parameters of the distributions determined by the error model Ω_*c*_. The probabilities *p_d_*, *p_poisson_* and *p_NB_* are calculated based on distributions

(4) $$\{\begin{array}{cc} r_{c}∼NB(e) & \text{ amplified }\\ r_{c}∼Poisson(0.1) & \text{ dropout},\; \end{array}$$

where *e* is the expected expression magnitude estimated as a median observed magnitude in cells where the gene is amplified.

### Model-based analysis of single-cell transcriptomics (MAST)

The MAST method has originally been designed for quantitative polymerase chain reaction-based fluidigm single-cell gene expression assay, but it can also be used for scRNA-seq data [8]. The theoretical basis of MAST lies in linear model fitting and likelihood ratio testing. MAST analysis consists of preprocessing, fitting a hurdle model and calculating the test statistics. The rate of expression (how many cells express the gene) and the level of expression for the expressed cells are modeled conditionally independently for each gene *g*.

Let $Z_{gc}\in \{0,1\}$ denote if gene *g* is expressed in cell *c* (*Z_gc_* = 1) or not (*Z_gc_* = 0), and let *Y_gc_* be the gene expression level when *Z_gc_* = 1. MAST fits a logistic regression model for *Z* and a Gaussian linear model for Y|Z=1:

(5) $$logit(P(Z_{gc}=1))=X_{c}\beta_{g}^{D}\text{ and }Y_{gc}|(Z_{gc}=1)∼N(X_{c}\beta_{g}^{G},\sigma_{g}^{2}),$$

where *X_c_* is the design matrix. The regression coefficients of both components are regularized. In the discrete case the regularization is done using a Bayesian approach in R-package arm. In the continuous case the variance parameter is regularized by cellular detection rate (CDR) defined as:

(6) $$CDR_{c}=\frac{1}{N}\sum_{g=1}^{N}Z_{gc},$$

where *N* is number of measured genes. Differential expression is determined using the likelihood ratio test.

### Differential expression analysis for sequence count data (DESeq)

The DESeq is a widely applied method originally developed for bulk RNA-seq data. It is based on a NB model, with mean and variance linked by local regression [9].

In DESeq, count *K_gj_* of gene *g* in sample *j* is modeled with NB distribution $K_{gj}∼NB(\mu_{gj},\sigma_{gj}^{2})$. Parameters *μ_gj_* and $\sigma_{gj}^{2}$ are not known and need to be estimated from the data. When comparing two groups (*A* and *B*) to each other, terms

(7) $$K_{Ag}=\sum_{j\in A}K_{gj}\text{ and }K_{Bg}=\sum_{j\in B}K_{gj}$$

are used. These variables are also expected to follow NB distributions $K_{Ag}∼NB(\mu_{Ag},\sigma_{Ag}^{2})$, *K_Bg_* similarly, and the parameters *μ_Ag_* and $\sigma_{Ag}^{2}$ can be derived from those of *K_gj_*. Let us denote the probability that $K_{Ag}=a$ and $K_{Bg}=b$ by *p*(*a*, *b*). Then the *P*-value *p_g_* of observed *K_Ag_* and *K_Bg_* is defined as

(8) $$p_{g}=2\cdot \frac{\sum_{\begin{array}{l} a+b=K_{Sg}\\ fc(a,b)\leq fc(K_{Ag},K_{Bg}) \end{array}}p(a,b)}{\sum_{a+b=K_{Sg}}p(a,b)},$$

where $K_{Sg}=K_{Ag}+K_{Bg}$ is the total sum of counts for gene *g*. Function *fc* denotes fold change

(9) $$fc(x,y)=\frac{x/s_{A}}{y/s_{B}},$$

where $s_{A}=\sum_{j\in A}s_{j}$ and, correspondingly, $s_{B}=\sum_{j\in B}s_{j}$.

### Linear models for microarray and RNA-Seq data (Limma)

The LIMMA method was originally designed for gene expression microarray data, but has recently been extended to RNA-seq data. In case of RNA-seq data, Limma uses Voom preprocessing [18]. Limma is based on linear modeling and it has shown good performance in previous comparison studies on bulk RNA-seq data [5, 6].

Limma considers gene-wise linear models

(10) $$E(y_{g})=X\alpha_{g}\text{ and }Var(y_{g})=W_{g}\sigma_{g}^{2},$$

where *y_g_* is a vector of expression values from different samples, *X* is a design matrix, *α_g_* is a coefficient vector and *W_g_* is a known weight matrix. The variable that describes possible differences between test groups is

(11) $$\beta_{g}=C^{T}\alpha_{g},$$

where *C* is a contrast matrix. The linear model is fitted to the responses to obtain coefficient estimators ${\hat{\alpha }}_{g}$ and estimators $s_{g}^{2}$ of $\sigma_{g}^{2}$. Contrast estimator is defined as ${\hat{\beta }}_{g}=C^{T}{\hat{\alpha }}_{g}$ with estimated covariance matrices

(12) $$Var({\hat{\beta }}_{g})=C^{T}V_{g}Cs_{g}^{2},$$

where *V_g_* is unscaled covariance matrix. Some assumptions about the distributions of ${\hat{\beta }}_{g}$ and $s_{g}^{2}$ are made so that ordinary t-statistic

(13) $$t_{gj}=\frac{{\hat{\beta }}_{gj}}{s_{g}\sqrt{v_{gj}}}$$

follows an approximate t-distribution with *d_g_* degrees of freedom. The term *v_gj_* is the *j*th diagonal element of $C^{T}V_{g}C$. Instead of regular t-statistic, Limma uses modified t-statistic

(14) $${\tilde{t}}_{gj}=\frac{{\hat{\beta }}_{gj}}{{\tilde{s}}_{g}\sqrt{v_{gj}}},$$

where

(15) $${\tilde{s}}_{g}^{2}=\frac{d_{0}s_{0}^{2}+d_{g}s_{g}^{2}}{d_{0}+d_{g}}.$$

Here a prior estimator $s_{0}^{2}$ and degree of freedom *d*_0_ are estimated from the data using empirical Bayes approach. If $d_{0}=0$, the modified *t*-test (14) is the ordinary *t*-test.

### Reproducibility-optimized test statistic (ROTS)

The ROTS is the only method among the tested ones that does not have any single-cell or sequencing-specific functions. ROTS optimizes the parameters among a family of modified t-statistics by maximizing the reproducibility of the detections across bootstrap samples. It has previously been shown to perform well in gene expression microarray and bulk RNA-seq data as well as in mass spectrometry-based proteomics data [11–13].

ROTS maximizes the scaled reproducibility

(16) $$\frac{R_{k}(d_{\alpha })-R_{k}^{0}(d_{\alpha })}{s_{k}(d_{\alpha })}$$

over the parameters $\alpha =(\alpha_{1},\alpha_{2})$ and *k*; $\alpha_{1}\in \left[0,\infty \right),\alpha_{2}\in \left\{0,1\right\}$, and *k* defines the top list size. The denominator $s_{k}(d_{\alpha })$ is the estimated standard deviation of the bootstrap distribution of the observed reproducibility $R_{k}(d_{\alpha })$. The term $R_{k}^{0}(d_{\alpha })$ corresponds to reproducibility of random data. It is calculated as the average reproducibility over randomized data sets, which are permuted from the real samples. The reproducibility is defined as

$$R_{k}(d_{\alpha })=\frac{1}{B}\sum_{b=1}^{B}R_{k}^{\left(b\right)}(d_{\alpha })$$

(17) $$=\frac{1}{B}\sum_{b=1}^{B}\frac{\#\{g|(r_{g}(\alpha ,D_{1}^{\left(b\right)})<k,r_{g}(\alpha ,D_{2}^{\left(b\right)})<k)\}}{k},$$

where *B* is the number of bootstrap rounds (two data sets $D_{1}^{b}$ and $D_{2}^{b}$ are generated in each round *b*), # denotes size of a set, and function *r_g_* returns the rank of a gene (or other element) *g* when test statistic $d_{\alpha }$ is used. The test statistic is a *t*-test-like statistic

(18) $$d_{\alpha }(g)=\frac{\left|\bar{x_{g}}-\bar{y_{g}}\right|}{\alpha_{1}+\alpha_{2}s_{g}},$$

where $\bar{x_{g}}$ and $\bar{y_{g}}$ are the averages of gene *g* over the samples in group *x* and *y*, respectively. The term *s_g_* is the standard error.

## Test design

### Data sets

The five methods were tested on three different scRNA-seq data sets with the objective of determining DE genes between different types of cells.

The first scRNA-seq data set, originated from a study by Islam *et al*. [14], is available in GEO database under accession number GSE29087 (accessed 13 November 2015). The count matrix available in GEO was used in the analysis. For validation of the results, the data set by Moliner *et al*. [15] was used, similarly as by Kharchenko *et al*. [7]. The .CEL files were available at http://carlosibanezlab.se//Data/Moliner_CELfiles.zip (accessed 30 October 2015). The original .CEL files were preprocessed using the Bioconductor package affy with Robust Multi-array Average (RMA) normalization. Only the genes appearing in both data sets were used in this study. Top 1000 DE genes were detected from the validation data using the Bioconductor package Limma.

The second scRNA-seq data set came from the study by Kowalczyk *et al*. [16]. The count matrix for mouse strain DBA that was used in this study is available in GEO under accession number GSE59114 (accessed 16 December 2015). The data consist of long-term hematopoietic stem cells (LTHSC). The data set is referred to here as LTHSC data set.

The third data set by Björklund *et al*. [17] contains innate lymphoid cell (ILC) data from human tonsil. The data set is available in GEO under accession number GSE70580 (accessed 13 April 2016). Only cell types ILC1, ILC2 and ILC3 were used in this study. This data set is referred to as ILC data set.

In each analyzed scRNA-seq data set, genes that were never expressed were filtered out. With SCDE, MAST, DESeq and Limma, we followed the instructions of the respective authors for data preprocessing and filtering. With ROTS we performed Trimmed Mean of M values normalization [4] before performing the differential expression analysis.

### Receiver operating characteristic performance, precision, recall and false positives

Our first comparison setting used the comparison design and data sets from the study by Kharchenko *et al*. [7]. More specifically, the single-cell data set by Islam *et al*. [14] was used to detect DE genes between 48 mouse embryonic stem cells and 44 mouse embryonic fibroblast cells. The five tested methods were then compared in terms of their area under the receiver operating characteristic (ROC) curve (AUC) using bulk expression measurements from Moliner *et al*. as a gold standard [15]. The AUC values were calculated with the R package pROC using the output of each method (absolute values of the test statistics or *P*-values) as predictor, and a binary vector, which indicates whether a gene belongs to the top 1000 validation genes, as response. The AUC values were compared between the five methods by *P*-values computed using bootstrap method.

Our second comparison setting used the relatively large single-cell data set by Kowalczyk *et al*. [16], which enabled us to also systematically assess the effect of the number of cells on the performance of the different methods. In these data, we compared LTHSC from old (20 months) and young (2–3 months) mice. After excluding cells with very low expression rate (sum of all counts < 10 000), a total of 135 cells from the old mice and 89 cells from the young mice remained in the data. To investigate the precision and recall of the methods when the number of cells per group was decreased in the LTHSC data, detections from the full data were considered as the gold standard. We used subsets of 70, 50, 30 and 10 cells per group. To avoid seemingly low or high precision and recall values occurring only owing to random selection of cells into subsets, the random selection was repeated 10 times per group size. Let *DE_full_* denote the set of detected DE genes in the full data set, and *DE_subset_* the set of detected DE genes in a subset of the data. The precision was defined as

(19) $$precision(DE_{full},DE_{subset})=\frac{\#(DE_{full}\cap DE_{subset})}{\#DE_{subset}},$$

where ∩ denotes the intersection between two sets. If $\#DE_{subset}=0$ then the precision value was set as missing. Recall was defined as

(20) $$recall(DE_{full},DE_{subset})=\frac{\#(DE_{full}\cap DE_{subset})}{\#DE_{full}}.$$

To estimate the rate of false-positive detections, we generated mock comparisons from the 135 LTHSC cells of the old mice by randomly dividing the cells into two non-overlapping groups that represent two cell populations. These two fake populations were compared with each other and the procedure was repeated 10 times. Because all the cells were from the same population, there should not be any real differences. Therefore, any possible detections would be considered false positive.

To consider data from other organisms than mouse, the third comparison was applied to the human data set by Björklund *et al*. [17]. Similarly as in original publication [17], we compared ILC3 cells to the other ILC cells; the case group consists of 308 ILC3 cells, and the control group consists of 266 ILC1 or ILC2 cells. Similar evaluations were carried out as in the second comparison setting. Because of the large size of the data, we tested also the subset size of 150 per group in addition to the sizes of 70, 50, 30 and 10 used also in the comparison for LTHSC data. The mock comparison for this data set was carried out by comparing two groups both consisting of 70 ILC3 cells. Similarly, as in the LTHSC comparison, the random selection for subsets and mock data sets was repeated 10 times in this data set.

## Results and discussion

### ROC performance with bulk benchmark

**Figure 1:**
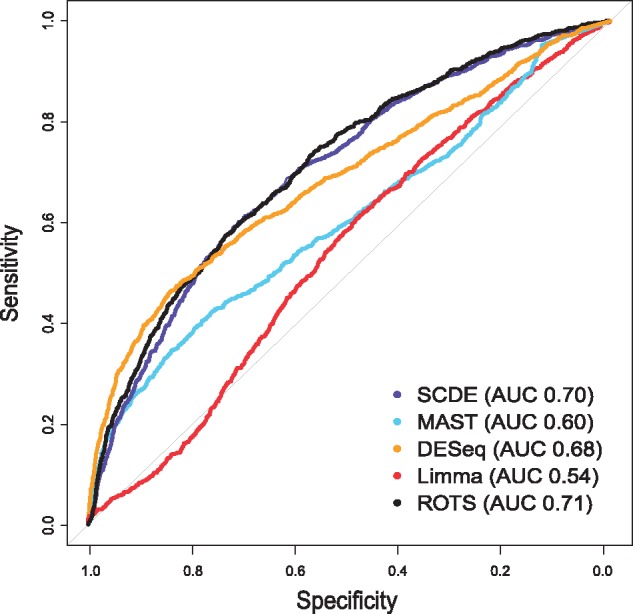
ROC curves of the five differential expression methods using a bulk benchmark. Sensitivity is shown as a function of specificity with the five statistical methods in the scRNA-seq data by Islam *et al.* [14]. Bulk measurements from the study by Moliner *et al.* [15] were used as the gold standard, following the comparison design by Kharchenko *et al.* [7]. Areas under the ROC curves (AUC) are shown in the parentheses. A colour version of this figure is available at BIB online: https://academic.oup.com/bib.

The ROC curves of the different methods are shown in Figure 1 together with the corresponding AUC values. In this comparison, SCDE (AUC 0.70) and ROTS (AUC 0.71) produced the highest AUC values, followed by DESeq (AUC 0.68). The single-cell-specific tool MAST performed significantly worse in this comparison (AUC 0.60), while Limma showed the overall lowest performance (AUC 0.54). The *P*-values for comparing the AUC values are shown in Table 1.

**Table 1:** *P*-values of the pairwise differences between areas under the ROC curves in the Islam *et al.* data [14] calculated using bootstrap method

Method	SCDE	MAST	DESeq	Limma	ROTS
SCDE	–	1.1e-12	0.14	<2.2e-16	0.44
MAST	1.1e-12	–	5.4e-08	1.0e-05	1.6e-15
DESeq	0.14	5.4e-08	–	<2.2e-16	0.03
Limma	<2.2e-16	1.0e-05	<2.2e-16	–	<2.2e-16
ROTS	0.44	1.6e-15	0.03	<2.2e-16	–

It is worth noting that the results were not identical to those reported by Kharchenko *et al*. [7], which is likely owing to different preprocessing of the data sets. Especially the benchmark validation data set is sensitive to differences in preprocessing because of the small number of samples. Nevertheless, despite the influence of preprocessing, the relative performance between the methods remained similar.

### Number and reproducibility of detections when reducing the number of cells

**Figure 2:**
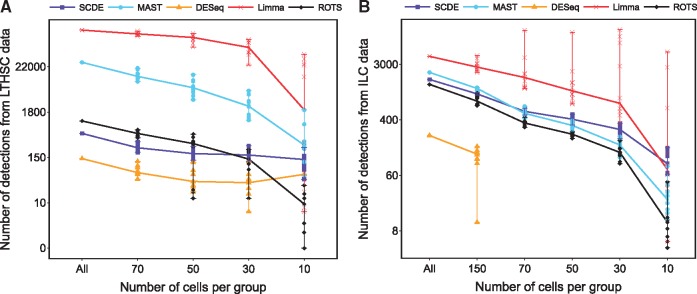
Number of DE genes with the five methods in (**A**) LTHSC data by Kowalczyk *et al.* [16] and (**B**) ILC data by Björklund *et al.* [17]. The number of detections is shown for the full data (All) and for the reduced data with decreasing number of cells per group. Different points in reduced sample sizes correspond to 10 randomly sampled subsets and the median numbers are connected with a line. DESeq could not be used in reduced ILC data with <150 cells per group. The y-axis is in log scale for the clarity of illustration. A colour version of this figure is available at BIB online: https://academic.oup.com/bib.

Figure 2 shows the numbers of DE genes (FDR <0.05) detected with the different methods in the full LTHSC and ILC data sets and in random subsets of sizes 10–150 cells per group, repeated 10 times for each size and data set. As expected, the number of detections decreased when the number of cells per group decreased, but the decrease was not steep before moving toward the 10 cells per group. From both data sets (LTHSC and ILC), Limma detected the largest number of DE genes and DESeq the lowest number; SCDE and ROTS had similar numbers of significant findings that were between the two extremes. In ILC data set, MAST behaved similarly to SCDE and ROTS, but in LTHSC data set it detected more DE genes than SCDE and ROTS. Notably, Limma tended to detect almost all of the genes as DE in the LTHSC data and, therefore, it was excluded from further comparisons in that data. DESeq threw errors with almost all sample sizes in ILC data, and so it was excluded from further comparisons in that data. For large data sets (>50 cells per group), the MAST algorithm threw warnings concerning convergence. However, the performance of MAST was still reasonably good, so these warnings were not considered critical.

**Figure 3:**
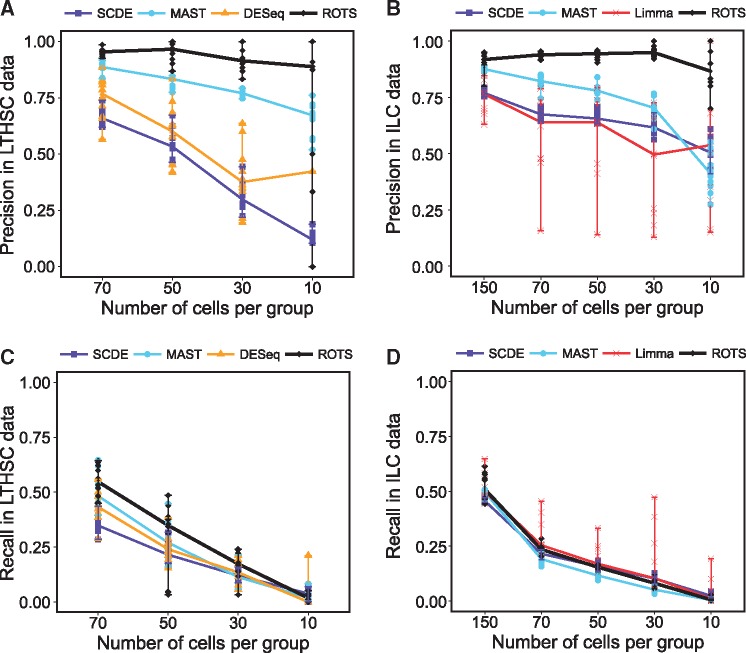
Precision and recall of the five methods in two data sets: precision (**A**) in LTHSC data by Kowalczyk *et al.* [16] and (**B**) in ILC data by Björklund *et al.* [17], and recall (**C**) in LTHSC data and (**D**) in ILC data. The values were calculated using DE genes in the full data as gold standard. Limma found almost everything as significant in all comparisons from the LTHSC data set; hence, for clarity of illustration it was excluded from those figures. In the precision figure of LTHSC data set, nine values were missing for DESeq with 10 cells per group owing to undefined values generated from zero detections. In the ILC data set, DESeq did not work with subsets of < 150 cells per group and it was therefore excluded from those figures. A colour version of this figure is available at BIB online: https://academic.oup.com/bib.

Next, we measured the precision and recall of the detections using the detections from the full data of 135 + 89 LTHSC cells and 308 + 266 ILC cells as the gold standard. The precision and recall values in both data sets for different methods and different numbers of cells per group are illustrated in Figure 3. When the number of cells was 70 per group, all of the tested methods had relatively high precision values (sampling medians ranged from 0.66 by SCDE to 0.95 by ROTS in LTHSC data and from 0.64 by Limma to 0.94 by ROTS in ILC data, see Figure 3A and B, respectively). For MAST and ROTS, the precision values remained high in both data sets when sample size was reduced (excluding MAST for 10 cells in ILC data set). SCDE clearly performed better in ILC data set than in LTHSC data set in both general precision level and sample size effect. Limma was excluded from LTHSC data set analysis because it detected almost everything as significant; in ILC data set analysis, its performance was the most modest among the tested methods. DESeq was excluded from ILC data set analysis because it could not be used with most of the reduced data sets; in analysis of LTHSC data set, it was among the worst-performing methods together with SCDE. With small sample size subset (10 cells per group) in LTHSC data set, DESeq produced detections only in 1 of the 10 randomly sampled subsets.

The recall values were systematically lower than the precision values for all the methods (Figure 3). The median recall values for random samplings were <0.55 for all the methods and all sample sizes in both data sets (Figure 3C and D). The recall values rapidly decreased for all the methods when the number of cells was decreased; at 10 cells per group, all the methods had sampling median close to 0. The lower levels of recall values compared with precision values is an expected outcome for two reasons. First, with smaller number of cells (subset versus full data), the differences between the two groups have to be larger to be significant. Second, when fewer cells are analyzed there are more genes that are never detected and therefore excluded.

### False-positive findings

**Figure 4:**
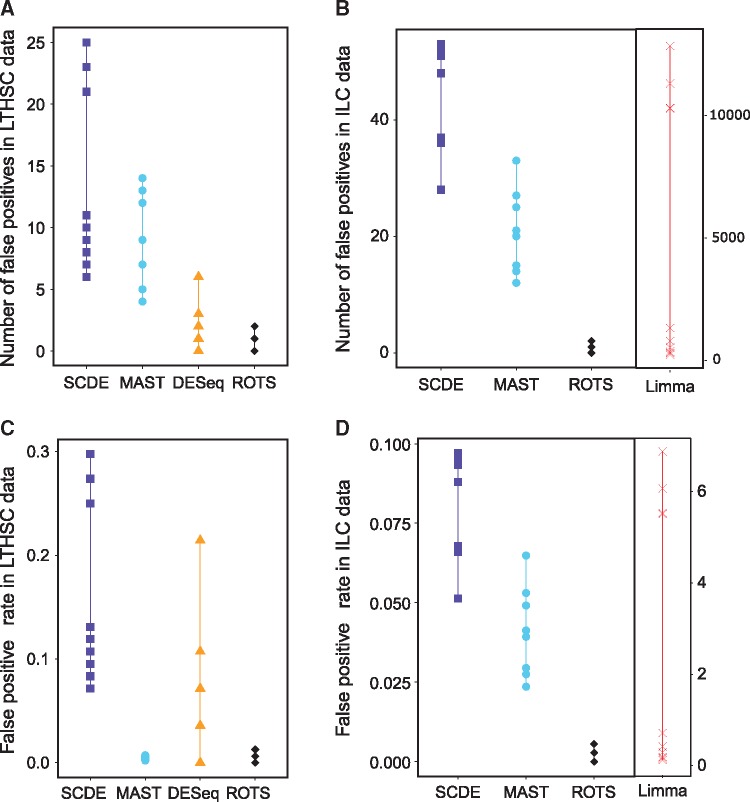
The number and rate of false positives on the basis of mock comparisons generated using two data sets: number of false positives (**A**) in LTHSC data by Kowalczyk *et al.* [16] and (**B**) in ILC data by Björklund *et al.* [17], and false-positive rate (**C**) in LTHSC data and (**D**) in ILC data. In both mock comparisons, DE genes were detected between two artificially constructed subsets of cells from the same population, in which no significant detections were expected. The points correspond to 10 randomly generated mock data sets. Limma could not be used with LTHSC data and DESeq could not be used with ILC data. In ILC data, Limma had high number of false positives and false positive rate compared with the other methods and therefore it was visualized separately. When there are <10 points visible, some of the points are overlapping. A colour version of this figure is available at BIB online: https://academic.oup.com/bib.

In Figure 4 the absolute numbers and rates of false positives are illustrated for LTHSC and ILC data sets. The false-positive rate was defined as the number of false-positive findings scaled by the median number of detected DE genes from the real comparison with similar number of cells (70 cells per group). Again, Limma was excluded from LTHSC analyses and DESeq from ILC analyses. MAST and ROTS made relatively few false-positive detections in both data sets. The median false-positive rate of MAST was 0.005 in LTHSC data set and 0.045 in ILC data set. The respective medians of ROTS were 0.006 and 0.001. SCDE performed better than Limma, but not as well as MAST and ROTS. With Limma the number and rate of false positives were high compared with the other methods. The median false-positive rate of Limma was 0.57, whereas all the other methods had median false-positive rate <0.1. The false-positive rates of DESeq varied more than those of MAST or ROTS. Overlaps between the false-positive findings across the different random subsamplings were low for all the methods, indicating that none of the tested methods is biased in detecting specific genes as DE.

### Similarity between the methods

Finally, we compared the overlaps of the detections between the methods in the three different data sets by extracting ∼10% of the most DE genes from the results of each method (600 genes in the Islam *et al*. data [14], 2000 genes in the Kowalczyk *et al*. data [16], 3500 genes in the Björklund *et al*. [17] data). Overall, the overlaps between the methods were relatively low (always <70%) and none of the tested methods showed systematically higher overlap than the other methods in all three data sets considered. The overlaps between the methods are listed in Table 2.

**Table 2:** Overlap (%) of top ∼10 % significant detections between the different methods in the data by Islam et al. [14], the LTHSC data by Kowalczyk et al. [16] and ILC data by Björklund et al. [17]

Method	SCDE	MAST	DESeq	Limma	ROTS
Data set by Islam et al. (mouse)					
SCDE	100	27	39	9	47
MAST	27	100	55	1	67
DESeq	39	55	100	5	62
Limma	9	1	5	100	3
ROTS	47	67	62	3	100
LTHSC data set (mouse).					
SCDE	100	24	35	10	39
MAST	24	100	18	2	36
DESeq	35	18	100	14	26
Limma	10	2	14	100	3
ROTS	39	36	26	3	100
ILC data set (human)					
SCDE	100	57	30	57	56
MAST	57	100	29	62	60
DESeq	30	29	100	25	24
Limma	57	62	25	100	58
ROTS	56	60	24	58	100

The modest overlaps between top detections from different methods indicate that the methods perform differently from one another.

### Running time

Among the tested methods, Limma is the fastest to run; MAST and DESeq are also relatively fast, whereas ROTS and SCDE are more computationally intensive. The time-consuming steps of SCDE and ROTS are fitting an error model and permuting the samples for bootstrapping, respectively. The remaining three methods, MAST, DESeq and Limma, do not include any heavily time-consuming steps. Details about running times with regard to different sizes of input data are available in Table 3.

**Table 3:** Running time (seconds) of each method with different sizes of input data

Methods	**Number of cells per group**				
	**All**	70	50	30	10
SCDE	17 962.8	10 200.5	5896.2	2113.8	322.8
MAST	135.2	110.7	100.9	84.6	61.5
DESeq	21.4	14.1	11.6	8.0	3.5
Limma	7.1	4.9	4.0	3.0	1.9
ROTS	805.8	624.0	538.4	426.6	293.6

The analyzed data are from LTHSC data by Kowalczyk et al. [16].

The times were recorded using system.time function in R. The user times listed in the table correspond to a system with Intel i7-4790 processor (3.60 GHz) and 32 GB of RAM. For the sake of comparability, only one core was used for the runs in Table 3. Otherwise SCDE includes an option to use multiple cores and the algorithm of ROTS is also suitable for parallel computing.

**Table 4:** Summary of the performance of the methods

Feature	SCDE	MAST	DESeq	Limma	ROTS
Designed for	scRNAseq	scRNAseq	Bulk RNAseq	Bulk RNAseq	General
Number of significant findings	Medium	High	Low	Very high	Medium
False positive rate	Medium	Low	Unstable	High	Very low
Reproducibility	Unstable	High	Low	Low/unstable	High
Running time	Very slow	Medium	Fast	Fast	Slow

## Conclusions

We have performed a systematic comparison of five different statistical methods to detect DE genes between single-cell populations. The single-cell-specific methods SCDE and MAST performed differently from each other. SCDE performed well in the first comparison setting in the Islam *et al*. data [14], but was among the worst performing methods in LTHSC and ILC data sets. MAST had low AUC in the Islam *et al*. data, but it performed well in both LTHSC and ILC data sets. Based on our comparisons, DESeq and Limma without any modifications are not suitable for scRNA-seq data analysis, and yet, they have performed well in the context of bulk RNA-seq data [5, 6]. ROTS is the most general method among the tested methods, as it is not designed specifically for sequencing data (bulk or single-cell). However, it performed well in all the comparisons in this study.

MAST detected much more DE genes than the other tested methods (excluding Limma, which detected almost everything from LTHSC data set), whereas DESeq yielded the smallest number of detections. SCDE and ROTS found similar numbers of DE genes and the numbers were between the two extremes. Overall the relative differences between the methods were similar in the LTHSC and ILC data sets. It is based on the application whether a high or low number of detections is more favorable. The conclusions are summarized in Table 4.

In addition to statistical properties, other features are also relevant when deciding which method to use [6, 19]. For example, SCDE, DESeq, Limma and ROTS require count data, whereas MAST prefers transcripts per million [20]. DESeq, Limma and ROTS are rather user-friendly and SCDE has a detailed tutorial available (http://hms-dbmi.github.io/scde/tutorials.html). All the methods are available as R packages.

Overall, our comparisons in three diverse data sets showed marked differences between the five tested methods in terms of the number of detections as well as their sensitivity and specificity, but did not reveal systematic benefits of the single-cell-specific methods in general. Instead, our previously introduced reproducibility-optimization method ROTS showed good performance in all comparisons even without any single-cell-specific modifications.

Key Points

- Selection of the method had a large impact on the results.
- Single-cell-specific methods did not have systematically better performance than other methods.
- Methods developed originally for bulk RNA-seq, DESeq and Limma, were not suitable for analyzing scRNA-seq data.
- ROTS performed well in all the comparisons and MAST had good statistical properties (false positives, precision and recall) as well.

## Supplementary data

Supplementary data are available online at http://bib.oxfordjournals.org/.

## Supplementary Material

Supplementary Data

## Notes

**Maria K. Jaakkola** is a doctoral student in applied mathematics at University of Turku, Finland. Her research topics are related to bioinformatics and pathway analysis under supervision of adjunct professor Laura L. Elo.

## Notes

**Fatemeh Seyednasrollah** is a doctoral student in bioinformatics at University of Turku, Finland. She is doing research in areas related to bioinformatics and computational biology under supervision of adjunct professor Laura L. Elo.

## Notes

**Arfa Mehmood** is a doctoral student in bioinformatics at University of Turku, Finland. She works with RNA sequencing and high-throughput data under supervision of adjunct professor Laura L. Elo.

## Notes

**Laura Elo** is adjunct professor in biomathematics and research director in bioinformatics at Turku Centre for Biotechnology, University of Turku, Finland. Her research group focuses on computational biomedicine and bioinformatics.
